# Development and validation of anthropometric prediction equations for estimation of lean body mass and appendicular lean soft tissue in Indian men and women

**DOI:** 10.1152/japplphysiol.00777.2013

**Published:** 2013-08-15

**Authors:** Bharati Kulkarni, Hannah Kuper, Amy Taylor, Jonathan C. Wells, K. V. Radhakrishna, Sanjay Kinra, Yoav Ben-Shlomo, George Davey Smith, Shah Ebrahim, Nuala M. Byrne, Andrew P. Hills

**Affiliations:** ^1^Institute of Health and Biomedical Innovation, Queensland University of Technology, Brisbane, Australia;; ^2^National Institute of Nutrition, Hyderabad, India;; ^3^Department of Non-communicable Disease Epidemiology, London School of Hygiene and Tropical Medicine, London, United Kingdom;; ^4^School of Social and Community Medicine, University of Bristol, Bristol, United Kingdom;; ^5^Childhood Nutrition Research Centre, University College London Institute of Child Health, London, United Kingdom;; ^6^South Asia Network for Chronic Disease, Public Health Foundation of India, New Delhi, India; and; ^7^Mater Mothers' Hospital, Mater Research and Griffith Health Institute, Griffith University, Brisbane, Australia

**Keywords:** lean body mass, appendicular lean soft tissue, anthropometry, prediction equation, Indian

## Abstract

Lean body mass (LBM) and muscle mass remain difficult to quantify in large epidemiological studies due to the unavailability of inexpensive methods. We therefore developed anthropometric prediction equations to estimate the LBM and appendicular lean soft tissue (ALST) using dual-energy X-ray absorptiometry (DXA) as a reference method. Healthy volunteers (*n* = 2,220; 36% women; age 18-79 yr), representing a wide range of body mass index (14–44 kg/m^2^), participated in this study. Their LBM, including ALST, was assessed by DXA along with anthropometric measurements. The sample was divided into prediction (60%) and validation (40%) sets. In the prediction set, a number of prediction models were constructed using DXA-measured LBM and ALST estimates as dependent variables and a combination of anthropometric indices as independent variables. These equations were cross-validated in the validation set. Simple equations using age, height, and weight explained >90% variation in the LBM and ALST in both men and women. Additional variables (hip and limb circumferences and sum of skinfold thicknesses) increased the explained variation by 5–8% in the fully adjusted models predicting LBM and ALST. More complex equations using all of the above anthropometric variables could predict the DXA-measured LBM and ALST accurately, as indicated by low standard error of the estimate (LBM: 1.47 kg and 1.63 kg for men and women, respectively), as well as good agreement by Bland-Altman analyses (**Bland JM, Altman D.** *Lancet* 1: 307–310, 1986). These equations could be a valuable tool in large epidemiological studies assessing these body compartments in Indians and other population groups with similar body composition.

lean body mass (LBM), the metabolically active compartment of the body, plays a central role in a number of physiologic processes (24). Muscle mass, which is a major component of LBM, is particularly important for insulin sensitivity and plays a protective role against chronic diseases, such as osteoporosis (31). Estimation of muscle mass and LBM during nutritional assessment, therefore, provides important insights.

MRI is considered to be a “gold standard” for evaluating skeletal muscle mass due to its high accuracy and lack of radiation to the subjects (18). However, MRI is expensive and not widely available for use in research and clinical practice. Dual-energy X-ray absorptiometry (DXA) is an attractive alternative approach to estimate LBM and skeletal muscle mass because of its good precision, less radiation exposure than other imaging techniques, such as computerized tomography (CT) scanning, and substantially lower cost than MRI. DXA can estimate appendicular lean soft tissue (ALST), which is used as a surrogate of skeletal muscle mass (11, 12), and the majority of the operative definitions of sarcopenia uses cut-points based on estimation of ALST by DXA (4, 7).

DXA, however, is not portable and is impractical for use in large-scale epidemiological studies. Anthropometry is one of the oldest techniques of body composition assessment, which has been validated by cadaver studies and other gold-standard methods (5, 21, 23). It offers distinct advantages of being simple, portable, noninvasive, and inexpensive. The use of anthropometry to assess body composition, therefore, continues to play an important role in clinical practice and in large population-based studies.

Anthropometric assessment of body composition relies on prediction equations derived from gold-standard methods. Commonly used prediction equations, including Durnin and Womersley's (6) and Jackson and Pollock's (9, 10) equations, have been developed in Caucasian populations. Population-specific prediction equations are, however, desirable due to ethnic differences in body composition (19). We, therefore, developed equations to predict LBM and ALST based on anthropometric variables using DXA as a reference method in a sample of Indian adults.

## PARTICIPANTS AND METHODS

The study was approved by the ethics committees of the National Institute of Nutrition, London School of Hygiene and Tropical Medicine, and Queensland University of Technology.

Healthy volunteers (*n* = 2,364) were enrolled in the study from two pre-established cohorts living around the city of Hyderabad, India. The first group of participants (*n* = 1,448; 32% women; age range: 18–23 yr) included members of a birth cohort established to assess the long-term impact of early nutrition supplementation provided to pregnant women and young children (Andhra Pradesh Children and Parents Study) (13). The second group included participants of the Hyderabad arm of the Indian Migration Study that was established to examine the association between rural to urban migration and cardiometabolic risk (*n* = 916; 46% women; age range: 21–79 yr). Of these, 108 participants (including 26 women who were pregnant) did not undergo DXA scanning. In addition, data on 36 participants were excluded due to poor scan quality. Finally, data on 2,220 participants who had outcome measurements done by anthropometry as well as DXA were included in the analyses. Demographic information was collected on all study participants using a standardized, interviewer-administered questionnaire.

### 

#### Anthropometric measurements.

These measurements were carried out by two trained investigators using standardized procedures (20). Weight was measured to the nearest 0.1 kg in light clothes without footwear using a digital scale (Seca, Birmingham, UK). Standing height was measured using a portable stadiometer (Leicester height measure; Chasmors, Camden, London, UK). Circumferences [mid-arm (MAC), calf, and hip] were measured to the nearest millimeter using a nonstretch narrow metal tape (metallic tape; Chasmors). MAC was measured at the midpoint between the tip of the acromion and olecranon process with the participant's arm flexed at 90°. Calf circumference was measured at the widest part of the lower leg. Hip circumference was measured at the widest part of the buttock. Skinfold thickness (SFT) was measured at four sites (biceps, triceps, subscapular, and suprailiac) to the nearest 0.1 mm using a Holtain caliper (Chasmors). SFT measurements were recorded three times, and the rest of the measurements were done twice. The average of the measured values for each was used in the analysis.

Body mass index (BMI) was calculated as weight (kg)/height (m)^2^. Corrected arm muscle area (CAMA; in cm^2^) was calculated using the MAC and triceps skinfold measurements with the following formula: CAMA = [MAC − (*π* × TSF)]^2^/4π − BA, where MAC is in centimeters, TSF = triceps SFT (in cm), and BA = correction for bone area, which was considered to be 10 cm for men and 6.5 cm for women (8). Anthropometric variables were also used to calculate body-fat percent using equations developed by Durnin and Womersley (6), because these equations are widely used in epidemiological studies to calculate the body fat and LBM (14, 16). Body-fat percent values using these equations were initially converted to fat mass (fat mass = body weight × percentage body fat), and the LBM values were then derived by subtracting the fat-mass values from the body weight (27).

#### DXA measurements.

A whole-body DXA scan was carried out for each participant using either the Hologic Discovery A model (Bedford, MA; 90% of scans) or Hologic 4500W (10% of scans) on the same day as anthropometry. The scanners were calibrated daily with a phantom, and their performance was monitored as per quality-assurance protocol. During the scan, participants were asked to lie supine on the scanning table with their arms at their sides. Pregnant women were excluded from the DXA scanning. Standard Hologic software options were used to define regions of the body (head, arms, trunk, and legs). ALST was calculated as the sum of bone-free lean tissue in arms and legs.

#### Statistical analyses.

All analyses were conducted using Stata version 11.2 (College Station, TX). SFT measurements were log transformed to reduce the skewness of the distribution. Participants were divided randomly into prediction (60%) and validation (40%) groups. To reduce the influence of outliers in the case of the anthropometric and DXA variables, extreme values below the first percentile and above the 99th percentile were adjusted and made equivalent to the first and 99th percentile, respectively. Participant characteristics in the prediction and validation groups were compared using Student's *t*-test. In the prediction data set, two sets of step-wise multiple linear regression analyses were performed to predict LBM and ALST using anthropometric variables as predictor variables. Anthropometric variables (weight; height; arm, calf, and hip circumferences; CAMA; and sum of four skinfolds) were entered in different combinations as predictors of LBM and ALST. The models were adjusted additionally for the DXA scanner and participant age. Coefficient of determination (adjusted *R*^2^), standard error of the estimate (SEE), and Akaike information criterion (AIC) (1) were used to evaluate the precision of the equations. Equations with a high *R*^2^, a small SEE, and the smallest AIC value were considered to be the best “fit” models.

The equations developed using the prediction set data were applied to the validation set data to calculate the predicted values of LBM and ALST. The predicted values were compared with the values measured by DXA using a paired sample *t*-test in the validation group. The pure error (PE) was calculated as the square root of the mean of squares of differences between measured and predicted values of the LBM and ALST. A smaller PE value indicated greater accuracy of the equation. The predicted and measured values were also compared using the Bland and Altman method (2a).

## RESULTS

Characteristics of participants in the prediction and validation groups are presented in Table 1. There were no significant differences in age and physical characteristics of men in the two groups, but women in the validation group were younger and lighter and had lower LBM and ALST compared with women in the prediction group.

**Table 1.** Characteristics of participants in the prediction and validation group

	Men							Women						
	Prediction Group			Validation Group				Prediction Group			Validation Group			
	*n*	Mean	sd	*n*	Mean	sd	*P*	*n*	Mean	sd	*n*	Mean	sd	*P*
Age, yr	851	30.1	14.7	570	30.2	14.5	0.86	481	34.7	14.3	318	31.9	14.0	0.01
Height, cm	851	166.0	6.2	570	166	6.33	0.51	481	152.6	5.4	318	152.1	5.6	0.18
Weight, kg	851	58.6	11.6	570	58.8	10.7	0.70	481	54.1	13.8	318	51.5	13	0.01
BMI, kg/m^2^	851	21.2	4.0	570	21.3	3.6	0.92	481	23.2	5.6	318	22.3	5.3	0.03
LBM, kg	851	44.84	6.39	570	45.16	6.17	0.35	481	33.56	6.01	318	32.52	5.45	0.01
ALST, kg	851	20.22	2.84	570	20.33	2.84	0.44	481	13.93	2.54	318	13.46	2.25	0.01

BMI, body mass index; LBM, lean body mass; ALST, appendicular lean soft tissue.

### 

#### Prediction and validation of LBM.

Table 2 shows the proposed prediction equations for estimation of LBM (kg). The simplest model with age, weight, and height as predictor variables (*Eq. 1*) explained ∼90% of the variation in LBM in the case of men and women. Addition of circumferences at hip, calf, and arm and/or sum of SFT measurements at four sites (*Eqs. 2–4*) resulted in improved adjusted *R*^2^, reduced SEE, and AIC values, indicating better predictive qualities of the models with added variables. An increase in the value of adjusted *R*^2^ (from 0.90 to 0.94 in men and 0.91 to 0.92 in women) and a decrease in SEE (from 1.92 to 1.47 kg in men and from 1.84 to 1.63 kg in women) from *Eq. 1* to *Eq. 4* was, however, marginal. Based on the AIC, *Eq. 4*, which had the lowest value, can be considered the best fit model.

**Table 2.** Proposed anthorpometric equations for estimation of LBM (kg)

	Predictor Variables	Sex	*n*	Proposed Equations	Adjusted *R*^2^	SEE	AIC
*Equation 1*	Height,^a^ weight^b^	M	851	Lean mass = −15.605 − (0.032 × age^c^) + (0.192 × height) + (0.502 × weight)	0.90	1.92	3530
		F	481	Lean mass = −13.034 − (0.018 × age) + (0.165 × height) + (0.409 × weight)	0.91	1.84	1953
*Equation 2*	Height, weight, circumferences^d^	M	851	Lean mass = −9.326 − (0.015 × age) + (0.207 × height) + (0.574 × weight) **+** (0.285 × arm circumference) + (0.182 × calf circumference) − (0.305 × hip circumference)	0.92	1.76	3382
		F	481	Lean mass = 3.191 − (0.013 × age) + (0.122 × height) + (0.581 × weight) − (0.093 × arm circumference) + (0.023 × calf circumference) − (0.188 × hip circumference)	0.91	1.76	1918
*Equation 3*	Height, weight, skinfold thickness^e^	M	851	Lean mass = 13.782 − (0.018 × age) + (0.064 × height) + (0.697 × weight) − (5.842 × logarithm of sum of 4 skinfolds)	0.94	1.57	3190
		F	481	Lean mass = 1.689 − (0.014 × age) + (0.120 × height) + (0.499 × weight) − (3.315 × logarithm of sum of 4 skinfolds)	0.92	1.68	1871
*Equation 4*	Height, weight, circumferences at 3 sites, skinfold thickness at 4 sites	M	851	Lean mass = 10.385 − (0.005 × age) + (0.103 × height) + (0.680 × weight) + (0.288 × arm circumference) + (0.130 × calf circumference) − (0.183 × hip circumference) − (5.278 × logarithm of sum of 4 skinfolds)	0.94	1.47	3081
		F	481	Lean mass = 10.632 − (0.009 × age) + (0.102 × height) + (0.592 × weight) + (0.055 × arm circumference) + (0.043 × calf circumference) − (0.158 × hip circumference) − (3.174 × logarithm of sum of 4 skinfolds)	0.92	1.63	1845

Adjusted *R*^2^, coefficient of determination; SEE, standard error of the estimate (in kg); AIC, Akaike's information criterion.

a Height (in cm);

b weight (in kg);

c age (in yr);

d circumferences at arm, calf, and hip (in cm);

e skinfold thickness measurements at biceps, triceps, and subscapular and suprailiac regions (in mm).

**Fig. 1.**
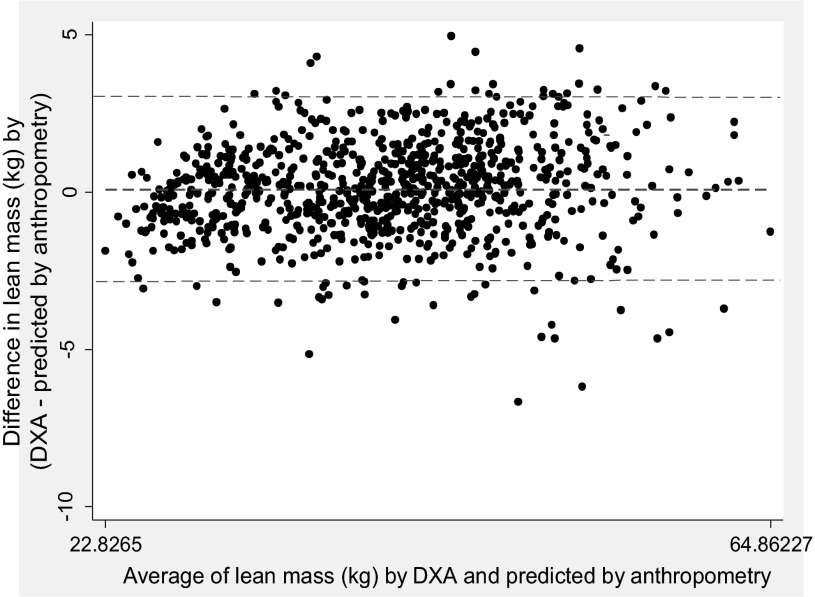
Bland-Altman plot of lean body mass (LBM; kg) estimates by dual-energy X-ray absorptiometry (DXA) and prediction equation based on sex-specific anthropometric variables. Equations used for prediction of LBM for men: LBM = 10.385 − (0.005 × age) + (0.103 × height) + (0.680 × weight) + (0.288 × arm circumference) + (0.130 × calf circumference) − (0.183 × hip circumference) − (5.278 × logarithm of sum of 4 skinfolds); for women: lean mass = 10.632 − (0.009 × age) + (0.102 × height) + (0.592 × weight) + (0.055 × arm circumference) + (0.043 × calf circumference) − (0.158 × hip circumference) − (3.174 logarithm of sum of 4 skinfolds).

The above four equations were then validated in the validation group participants. The mean differences between the LBM measured by DXA and *Eq. 1* based on age, height, and weight in the case of men and women were ∼0.28 kg and ∼0.02 kg, respectively (Table 3). Although this difference was statistically robust in the case of men, it was <1% of the mean LBM. The PE and limits of agreement were also relatively narrow. Inclusion of circumferences and SFTs as predictors in *models 2–4* reduced the difference between the measured and predicted LBM value, along with reduction in PE, and narowed the limits of agreement. The Bland-Altman plot comparing the LBM estimates measured by DXA and those predicted by *Eq. 4* also showed that the mean estimates by the two methods were similar with no evidence of bias (Fig. 1).

**Table 3.** Validation of anthropometric equations for estimation of LBM (kg) in the validation group

	Sex	*n*	Difference (DXA − Equation)^a^	sd	*P*^b^	Adjusted *R*^2^	Pure Error^c^	Limits of Agreement^d^	
*Equation 1*^e^	M	570	0.28	1.96	<0.01	0.90	1.96	−3.57	4.13
	F	318	0.02	1.64	0.83	0.91	1.64	−3.20	3.24
*Equation 2*^f^	M	569	0.23	1.91	<0.01	0.90	1.91	−3.52	3.98
	F	318	0.01	1.58	0.91	0.91	1.59	−3.09	3.11
*Equation 3*^g^	M	568	0.17	1.64	0.01	0.93	1.64	−3.04	3.39
	F	309	0.05	1.46	0.59	0.92	1.46	−2.82	2.91
*Equation 4*^h^	M	567	0.10	1.56	0.14	0.94	1.56	−2.96	3.16
	F	309	0.05	1.39	0.51	0.93	1.39	−2.68	2.79
Durnin-Womersley equation (6)	M	568	−4.32	2.18	<0.01	0.88	2.11	−8.61	−0.03
	F	309	−4.03	1.86	<0.01	0.89	1.76	−7.68	−0.39

a Difference in the estimates of LBM by dual-energy X-ray absorptiometry (DXA) − proposed anthropometric equation (in kg);

b *P*, based on paired *t*-test;

c pure error (in kg), calculated as square root of the mean of squares of differences between the LBM estimates by DXA and proposed equations;

d limits of agreement: 95% limits of agreement (mean difference ± 2 sd) by DXA and proposed equations calculated by the Bland-Altman technique (2a);

e *Eq. 1*: based on height and weight;

f *Eq. 2*: based on height, weight, and circumferences (arm, calf, and hip);

g *Eq. 3*: based on height, weight, and skinfold thickness at biceps, triceps, and subscapular and suprailic regions;

h *Eq. 4*: based on height, weight, circumferences (arm, calf, and hip), and skinfold thickness (biceps, triceps, and subscapular and suprailic regions).

When the values of LBM measured by DXA were compared with the LBM estimates derived by the commonly used Durnin and Womersley equation (6), the values predicted by this equation were substantially higher than the DXA estimates with a mean difference of 4.32 kg (2.18 SD) in men and 4.03 kg (1.86 SD) in women (Table 3). Although the values predicted by equations explained >88% of the variation in the DXA-measured LBM, the limits of agreement were wider than those using the equations developed in the present study.

#### Prediction and validation of ALST.

Table 4 shows the proposed prediction equations for estimation of ALST (kg). Compared with equations for estimation of LBM (Table 3), variation in ALST explained by these equations was lower, ranging from 0.78 by the simplest model (*Eq. 1*, including age, height, and weight) to 0.86 by *Eq. 4*, which additionally included circumferences and SFTs. SEE and AIC values were lowest with *Eq. 4* compared with simpler models proposed by *Eqs. 1–3*, indicating better prediction quality of the model compared with *Eqs. 1–3*. *Equation 3*, which included a derived index of CAMA in addition to height and weight, showed better prediction ability (lower SEE and AIC values and higher adjusted *R*^2^) compared with *Eq. 1*, indicating that inclusion of this index improved the precision of the estimate.

**Table 4.** Proposed anthorpometric equations for estimation of ALST (kg)

	Predictor Variables	Sex	*n*	Proposed Equations	Adjusted *R*^2^	SEE	AIC
*Equation 1*	Height,^a^ weight^b^	M	851	ALST = −13.432 − (0.0445 × age^c^) + (0.200 × weight) + (0.140 × height)	0.78	1.28	2842
		F	481	ALST = −9.852 − (0.028 × age) + (0.170 × weight) + (0.102 × height)	0.82	1.05	1420
*Equation 2*	Height, weight, circumferences^d^	M	851	ALST = −12.81 − (0.029 × age) + (0.211 × weight) + (0.153 × height) + (0.255 × calf circumference) + (0.141 × arm circumference) − (0.178 × hip circumference)	0.82	1.17	2687
		F	481	ALST = −2.658 − (0.023 × age) + (0.244 × weight) + (0.082 × height) + (0.087 × calf circumference) − (0.058 × arm circumference) − (0.102 × hip circumference)	0.84	1.01	1386
*Equation 3*	Height, weight, CAMA^e^	M	851	ALST = −16.270 − (0.037 × age) + (0.143 × weight) + (0.159 × height) + (0.087 × CAMA)	0.82	1.18	2696
		F	481	ALST = −10.818 − (0.027 × age) + (0.142 × weight) + (0.109 × height) + (0.051 × CAMA)	0.83	1.02	1394
*Equation 4*	Height, weight, circumferences, skinfolds^f^	M	851	ALST = −0.996 − (0.023 × age) + (0.274 × weight) + (0.090 × height) + (0.223 × calf circumference) + (0.143 × arm circumference) − (0.104 × hip circumference) − (3.163 × logarithm of sum of 4 skinfolds)	0.86	1.02	2452
		F	481	ALST = 1.609 − (0.021 × age) + (0.250 × weight) + (0.070 × height) + (0.098 × calf circumference) + (0.027 × arm circumference) − (0.085 × hip circumference) − (1.821 × logarithm of sum of 4 skinfolds)	0.86	0.94	1314

a Height (in cm);

b weight (in kg);

c age (in yr);

d circumferences at arm, calf, and hip (in cm);

e CAMA, corrected arm muscle area (in cm);

f skinfold thickness measurements at biceps, triceps, and subscapular and suprailiac regions (in mm).

**Fig. 2.**
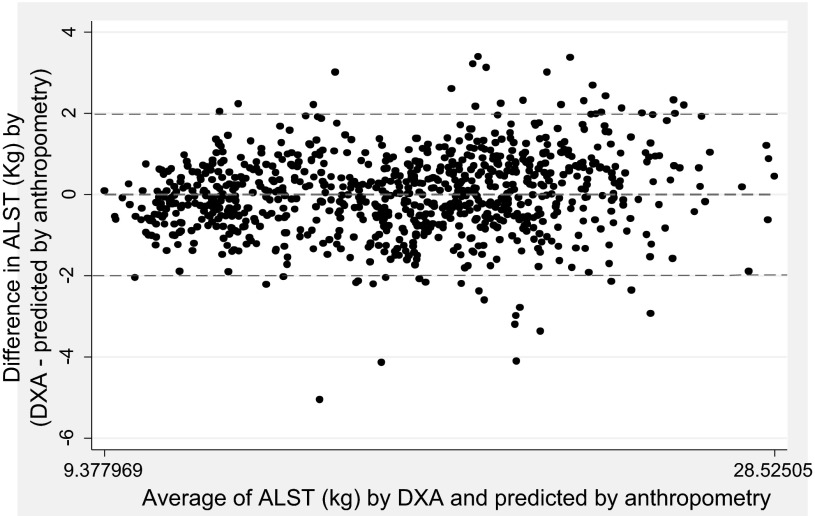
Bland-Altman plot of appendicular lean soft tissue (ALST; kg) estimates by DXA and prediction equation based on sex-specific anthropometric variables. Equations used for prediction of ALST for men: ALST = −0.996 − (0.023 × age) + (0.274 × weight) + (0.090 × height) + (0.223 × calf circumference) + (0.143 × arm circumference) − (0.104 × hip circumference) − (3.163 × logarithm of sum of 4 skinfolds); for women: ALST = 1.609 − (0.021 × age) + (0.250 × weight) + (0.070 × height) + (0.098 × calf circumference) + (0.027 × arm circumference) − (0.085 × hip circumference) − (1.821 × logarithm of sum of 4 skinfolds).

When the above models were applied to the validation group for prediction of ALST, the mean values of ALST estimates by DXA and prediction equations were similar, as the differences between the estimates by two methods were not statistically robust (Table 5). PE reduced from *Eq. 1* (men: 1.33 kg; women: 0.97 kg) to *Eq. 4* (men: 1.09 kg; women: 0.83 kg) as a result of additional variables added to the model used in *Eq. 4*. Limits of agreement were also narrower in the case of *Eq. 4* compared with *Eq. 1*. The Bland-Altman plot, comparing the two ALST estimates (measured by DXA and predicted by sex-specific *Eq. 4*), also showed that the mean estimates by the two methods were similar (Fig. 2).

**Table 5.** Validation of anthropometric equations for estimation of ALST (kg) in the validation group

	Sex	*n*	Difference (DXA − Equation)^a^	sd	*P*^b^	Adjusted *R*^2^	Pure Error^c^	Limits of Agreement^d^	
*Equation 1*^e^	M	570	0.05	1.33	0.36	0.78	1.33	−2.56	2.67
	F	318	−0.06	0.97	0.31	0.82	0.97	−1.96	1.85
*Equation 2*^f^	M	569	0.01	1.30	0.89	0.79	1.30	−2.53	2.55
	F	318	−0.04	0.93	0.44	0.83	0.93	−1.86	1.78
*Equation 3*^g^	M	570	0.00	1.20	0.96	0.82	1.20	−2.36	2.36
	F	318	−0.05	0.93	0.34	0.83	0.93	−1.87	1.77
*Equation 4*^h^	M	567	0.02	1.09	0.67	0.85	1.09	−2.11	2.15
	F	309	−0.02	0.83	0.69	0.86	0.83	−1.65	1.61

a Difference in the estimates of ALST by DXA − proposed anthropometric equation (in kg);

b *P* based on paired *t*-test;

c pure error (in kg), calculated as square root of the mean of squares of differences between the ALST estimates by DXA and proposed equations;

d limits of agreement: 95% limits of agreement (mean difference ± 2 sd) by DXA and proposed equations calculated by the Bland-Altman technique;

e *Eq. 1*: based on height and weight;

f *Eq. 2*: based on height, weight, and circumferences (arm, calf, and hip);

g *Eq. 3*: based on height, weight, and CAMA;

h Difference in the estimates of ALST by DXA − proposed anthropometric equation (in kg); *Eq. 4*: based on height, weight, circumferences (arm, calf, and hip), and skinfold thickness (biceps, triceps, and subscapular and suprailic regions).

## DISCUSSION

In the present study, we developed anthropometric prediction equations to estimate LBM and ALST in a large sample of healthy Indian adults using DXA as a reference method. Validation of the newly developed equations in a subsample showed that the equations predicted the LBM and ALST with high precision and low error. These equations, using simple, commonly used anthropometric measurements, could be a valuable tool in population-based studies assessing these important body compartments in Indians and other ethnic groups with similar body composition. However, use of these equations in other ethnic groups must be preceded by their validation in those population groups.

We used ALST as an indicator of muscle mass in this study. This is based on a concept that approximately three-fourths of total body muscle mass exists in the extremities and that appendicular lean tissue is primarily skeletal muscle (17). ALST, therefore, is commonly used as a surrogate of skeletal muscle mass, and DXA estimates of ALST have been validated using the skeletal muscle mass measurements by MRI and CT (3, 12, 28, 32). The guidelines on the diagnosis of sarcopenia also use cut-points based on ALST estimates (7). Moreover, cut-points based on ALST predicted physical disability in elderly men and women independent of other covariates, such as age, physical activity, and prevalent morbidity, in a longitudinal study from the United States (2). As access to DXA is limited in resource-poor settings, the anthropometric prediction equations developed in our study would be a valuable tool to detect low muscle mass in different population groups.

A number of researchers previously developed anthropometric prediction equations for estimation of body composition and validated them with cadaver studies (5, 21) and other criterion techniques, such as hydrodensitometry (6, 9, 10), MRI (26), and four compartment model (25, 29). However, the majority of these equations has typically calculated body-fat percentage, and only a few studies have specifically attempted prediction of LBM or skeletal muscle mass. For example, a study from the United States developed two anthropometric prediction equations to predict skeletal muscle mass in a multi-ethnic sample using MRI as a reference technique. A simpler equation using height and weight could estimate the skeletal muscle mass with a SEE of 2.8 kg, whereas a relatively complex equation that included skinfold-corrected limb circumferences showed improved prediction quality with a SEE of 2.2 kg (17). Equations for LBM and ALST estimation in the present study showed relatively higher precision (SEE < 2 kg and < 1.3 kg for estimation of LBM and ALST, respectively) compared with the study mentioned above, probably due to a larger sample size and homogeneous study sample. The SEEs of the equations in our study were also lower than those reported by equations in a study in young Indian men (*n* = 66) that used 24-h urinary creatinine excretion as a reference technique (15).

Results of our study are comparable with a study in Chinese adults that developed anthropometric prediction equations for estimation of ALST using DXA as a reference technique in a sample of 763 participants (30). The *R*^2^ of prediction models based on different combinations of height, weight, and limb circumferences in the Chinese study ranged from 0.90 to 0.93, with SEE ∼1.5 kg. These values are similar to those observed in our study, indicating that combinations of different anthropometric measurements can be used for accurate and precise estimation of LBM and ALST.

As the age range of study participants was wide, age was included as a predictor in all of the equations for prediction of LBM and ALST. Age had a negative association with LBM and ALST in all of the models, indicating an age-related reduction in the lean tissue. Among the anthropometric variables, limb circumferences had a positive association, whereas hip circumference had a negative association with these outcomes. This suggests that although arm and calf circumferences can be considered as indicators of muscle mass, hip circumference may be an indicator of gluetofemoral fat rather than the muscle mass in this region. As expected, the sum of SFTs was negatively associated with LBM and ALST, due to its close association with adiposity.

The equations using simple anthropometric variables of height and weight (*Eq. 1* in Tables 2 and 4) had lower prediction qualities (higher SEE, higher PE, and wider limits of agreement) compared with the equations with additional variables, such as hip and limb circumferences and SFTs. However, the contribution of these added variables to the explained variation in the measured LBM and ALST was only approximately 5–8% (Table 2). For example, *Eq. 1*, using age, height, and weight, explained 90% of the variation in measured LBM of men. Addition of hip and limb circumferences and SFTs to this equation (*Eq. 4*) increased the variation explained to 94%, indicating that equations based on simple measures of height and weight can provide reasonably precise estimates of LBM and ALST. The use of CAMA along with height and weight increased the adjusted *R*^2^ value and reduced the SEE (*Eq. 3* in Table 4) in prediction of ALST. Thus this model, with commonly measured anthropometric variables of arm circumference and TSF, can provide a fairly accurate estimation of ALST. However, the use of fully adjusted models that included height, weight, hip and limb circumferences, and SFT measurements alleviated the differences in the mean estimates of measured vs. predicted values of LBM and ALST (Figs. 1 and 2), showing excellent prediction quality of these models.

Durnin and Womersley's equations (6), which are commonly used in epidemiological studies to assess body composition using anthropometric measurements, did not predict LBM accurately in this study sample. The mean LBM values predicted by these equations were higher by ∼4.3 kg and ∼4.0 kg compared with DXA measures in men and women, respectively. On the other hand, the prediction equations developed in the present study using similar predictors (age, height, weight, and SFTs) could estimate the LBM with high accuracy with mean differences (DXA − equation) of 0.17 kg and 0.05 kg in men and women, respectively (*Eq. 3* in Table 3). The reasons for the lower prediction quality of the Durnin and Womersley's equation could be related to the fact that these equations were developed in Caucasian men and women using hydrodensitometry as a reference method.

The strengths of our study include a study sample representing a broad range of age and BMI and a large sample size that allowed development of equations with high prediction quality. To our knowledge, this is the first study from India to develop such equations using a precise technique of DXA as a reference method. Limitation of the study includes lack of data on SFT measurements at thigh and chest regions, which precluded comparison of our equations with some of the other commonly used equations, such as Jackson and Pollock's equations (9, 10).

In summary, in the present study, a number of sex-specific anthropometric prediction equations for estimation of LBM and ALST were developed and then cross-validated in an independent sample of healthy adults. Fully adjusted models that included hip and limb circumferences and SFTs, along with weight and height, predicted these outcomes with high accuracy. Simple models, including age, height, and weight, also predicted LBM and ALST with reasonably low prediction error. These equations based on commonly measured anthropometric variables used in our study can be used in a wide range of epidemiological studies collecting anthropometric data and could be a valuable tool in resource-poor settings. Additional validation studies are, however, needed to test their validity in different population groups.

## GRANTS

Funding for the study was provided by the Wellcome Trust, UK (grant number WT083707AIA).

## DISCLOSURES

The study sponsor had no role in the study design; in the collection, analysis, and interpretation of data; in writing the report; or in the decision to submit the paper for publication. Authors have no potential conflicts of interest.

## AUTHOR CONTRIBUTIONS

Author contributions: B.K. and H.K. conception and design of research; B.K. and H.K. performed experiments; B.K. analyzed data; B.K., N.M.B., and A.P.H. interpreted results of experiments; B.K. prepared figures; B.K. drafted manuscript; B.K., H.K., A.T., J.C.W., K.V.R., S.K., Y.B-S., G.D.S., S.E., N.M.B., and A.P.H. edited and revised manuscript; B.K., H.K., A.T., J.C.W., K.V.R., S.K., Y.B-S., G.D.S., S.E., N.M.B., and A.P.H. approved final version of manuscript.
